# Chokeberry Pomace as a Source of Polyphenols for Complexation with Plant-Based Proteins

**DOI:** 10.3390/molecules31173002

**Published:** 2026-08-27

**Authors:** Mirela Kopjar, Antun Jozinović, Iva Ostrun, Jurislav Babić, Anita Pichler

**Affiliations:** Faculty of Food Technology Osijek, Josip Juraj Strossmayer University of Osijek, Franje Kuhača 18, 31000 Osijek, Croatia; ajozinovic@ptfos.hr (A.J.); iostrun@ptfos.hr (I.O.); jbabic@ptfos.hr (J.B.); anita.pichler@ptfos.hr (A.P.)

**Keywords:** chokeberry pomace polyphenols, complexation, almond proteins, rice proteins, pea proteins, pumpkin-seed proteins

## Abstract

One way to utilize by-products from fruit production is the extraction of bioactive compounds and their subsequent complexation with appropriate carriers. Consequently, we used chokeberry pomace extract for complexation with plant-based proteins, namely almond, rice, pea, and pumpkin-seed protein matrices, which differ in their composition and protein content. The obtained complexes, as well as the chokeberry pomace extract and protein matrices, were evaluated for the concentration of individual polyphenols, total polyphenols, procyanidins, and antioxidant activity. Color parameters and IR spectra were also recorded. The results of this study emphasized the importance of the structure and properties of polyphenols and proteins, which are significant factors affecting polyphenol adsorption onto protein-based carriers. All proteins showed similar affinity for anthocyanins (69% for cyanidin-3-galactoside, and 23% to 29% for cyanidin-3-arabinoside). Rice proteins had the highest affinity for quercetin-3-rutinoside (87%), while pumpkin proteins showed the highest affinity for quercetin-3-galactoside (87%) and quercetin-3-glucoside (65%). Rice proteins also had the highest affinity for phenolic acids (67% for neochlorogenic acid, 81% for chlorogenic acid, and 48% for isochlorogenic acid). These results provide a good baseline for the formulation of dried protein-based complexes, based on chokeberry pomace, rich in polyphenols which can have possible uses in food products, to enhance their antioxidant potential.

## 1. Introduction

A considerable amount of by-products and waste remain after agro-food production. Even though they are considered waste, fruit by-products remain rich in nutrients and bioactive constituents, such as proteins, unsaturated fatty acids, minerals, dietary fiber, and various antioxidant compounds. This allows fruit by-products to be used in designing foods with functional properties, enhancing nutritional and health-promoting values of foods, as well as improving the technological and sensory characteristics of food products [1]. One of the targets of the UN Sustainable Development Goals is to accomplish zero-hunger by 2030 through the promotion and implementation of sustainable production and a circular economy [1,2]. One way to achieve this is through the production of upcycled foods, which are prepared from ingredients that would otherwise be wasted but are considered a sustainable answer to the food waste problem [2]. Pomace of chokeberry (*Aronia melanocarpa*) is a by-product of juice production, and is a rich, underutilized source of phenolic compounds, which are well known for their significant antioxidative and anti-inflammatory properties. Thus, it represents a good baseline for the preparation of upcycled foods. Generally, during juice-pressing from fruits, approximately 13% of the total polyphenols are transferred to the juice. The majority were phenolic acids (66%), followed by flavonoids (23%) and anthocyanins (10%) [3]. The most important polyphenols were anthocyanins (specifically cyanidin-3-galactoside and cyanidin-3-glucoside), phenolic acids (specifically chlorogenic and neochlorogenic acids), and flavonols (specifically quercetin and kaempferol glycosides) [3,4,5,6,7]. These data suggest that chokeberry pomace can be further used to prepare various extracts and to formulate bioactive ingredients, which are currently the focus of many researchers and can be considered as upcycled foods. Even though chokeberry pomace is a good source of polyphenols, which are known for their numerous health-promoting functions, their instability during processing, storage, and digestion cannot be overlooked. To address the issue of polyphenol instability and prepare bioactive ingredients, the choice of carriers is a crucial step.

Over the years, proteins have been proposed as effective biopolymers for the protection of polyphenols, with increasing focus on plant-based proteins due to population growth, rising consumer awareness of healthy food choices, and environmental concerns. Thus, plant-based proteins can also contribute to sustainability. Due to the presence of antinutritional factors, indigestible cell wall matrices, and specific protein structures, plant-based proteins have lower digestibility (75–80%) than animal proteins (90–95%) [8]. However, as they often contain, in addition to the protein fraction, nutritionally beneficial fibers and unsaturated fatty acids, exploration of improvements to their functionality and application in foods is increasing [9]. The utilization of plant-based proteins is increasing, and by 2030, it is estimated that 7.7% of the global protein market will contain plant-based foods, with a considerably higher market value than in 2020 [9]. Although the extraction of chokeberry polyphenols from pomace by different techniques is a well-studied field, this is not the case for complexation of its extracts (CPE), which results in adsorption or encapsulation of polyphenols onto different carriers. Complexation of CPE with plant proteins, followed by drying to obtain a dry, stable powder, has not been widely explored; therefore, we selected plant-based protein matrices that differ in their composition and protein content, namely, almond, rice, pea, and pumpkin-seed protein matrices as carriers for complexation with CPE. Some work in this field has been conducted using different carriers and techniques. Encapsulation of CPE in double emulsions has been investigated, showing that this system can be used for the protection of CPE polyphenols [10,11]. The inner water phase was prepared by adding sodium chloride and CPE to water, the oil phase by dispersing polyglycerol polyricinoleate in rapeseed oil, and the outer water phase from whey proteins. Catalkaya et al. [12] investigated spray-drying encapsulation of CPE with maltodextrin of different degrees of polymerization, in combination with gum Arabic, xanthan gum, or whey protein isolate, demonstrating that the choice of carrier has a major impact on encapsulation efficiency.

The aim of this study was to explore the possibility of formulating protein-based complexes. We selected four plant-based protein matrices (almond, rice, pea, and pumpkin-seed protein matrices) that differ in composition, structure, and protein content to examine which of them has the highest potential, based on these properties, to adsorb polyphenols from chokeberry pomace extract (CPE). This approach expands knowledge of CPE polyphenol adsorption on plant-based proteins, with the goal of formulating upcycled bioactive food ingredients, and can also enable the use of other fruit pomace for the same purpose. The resulting protein complexes were evaluated for total polyphenols, procyanidins, individual polyphenols, and antioxidant activity. Additionally, IR spectra were recorded to demonstrate the adsorption of CPE polyphenols on proteins, and color parameters were determined. Finally, the results of the investigated parameters were compared depending on the type of protein matrix.

## 2. Results

### 2.1. Polyphenol Profile and Antioxidant Activity

Firstly, the initial ingredients for the preparation of protein-based complexes were evaluated for polyphenols and antioxidant activity. The total polyphenol and procyanidin contents, as well as the antioxidant activities of the prepared chokeberry pomace extract (CPE) and selected protein matrices for encapsulation of CPE polyphenols, are presented in Table 1. CPE contained 0.769 g/L of total polyphenols and 5.910 mg/L of proanthocyanidins. Antioxidant activity values were 10.987 mmol TE/100 mL, 5.897 mmol TE/100 mL, 2.413 mmol TE/100 mL, and 3.891 mmol TE/100 mL for the CUPRAC, FRAP, DPPH and ABTS methods, respectively. Since the protein matrices were obtained from plant sources, they were also evaluated for polyphenols and antioxidant activity. In all four matrices, there was no detection of total polyphenols, while procyanidins were detected. The lowest procyanidin content was found in rice proteins (0.078 mg/g), followed by pumpkin and almond proteins (approximately 0.28 mg/g), and the highest content was found in pea proteins (0.480 mg/g). The protein matrices also exhibited antioxidant activity as determined by all methods. Using the CUPRAC method, antioxidant activity was approximately 4 mmol TE/100 g for pea and almond proteins, while for rice and pumpkin proteins these values were significantly lower, at 1.742 and 1.152 mmol TE/100 g, respectively. Applying the FRAP method, a different trend was observed. The highest antioxidant activity was recorded for pumpkin and almond proteins (0.725 mmol TE/100 g), followed by pea proteins (0.652 mmol TE/100 g), while rice proteins had the lowest antioxidant activity (0.187 mmol TE/100 g). With the DPPH method, similar antioxidant activity was observed for almond, rice and pumpkin proteins (approximately 0.44 mmol TE/100 g), while pea protein had a lower value (0.291 mmol TE/100 g). The highest antioxidant activity with the ABTS method was achieved for pumpkin proteins (0.338 mmol TE/100 g), followed by almond proteins (0.264 mmol TE/100 g), while the other two protein matrices had very low antioxidant activity (0.030 mmol TE/100 g).

Additionally, individual polyphenols in the initial ingredients were evaluated and are presented in Table 2. In CPE, two anthocyanins were detected, cyanidin-3-galactoside and cyanidin-3-arabinoside, in concentrations of 92.08 mg/L and 31.09 mg/L, respectively. Among flavonoids, quercetin-3-rutinoside, quercetin-3-galactoside, and quercetin-3-glucoside were found in concentrations of 127.72 mg/L, 109.94 mg/L, and 155.29 mg/L, respectively. The most abundant compounds in CPE were phenolic acids: neochlorogenic acid, isochlorogenic acid, and chlorogenic acid, which were present at concentrations of 1181.28 mg/L, 129.07 mg/L, and 731.05 mg/L, respectively. Additionally, catechin, epicatechin, and procyanidin B1 were determined at concentrations of 4.71 mg/L, 8.12 mg/L, and 5.59 mg/L, respectively. On protein matrices, anthocyanins, quercetin derivatives, and phenolic acids were not detected. Catechin, epicatechin, and procyanidin B1 were found on some protein matrices. The highest concentrations of these compounds were detected on pumpkin and almond protein matrices. On the almond protein matrix, catechin, and procyanidin B1 were determined at concentrations of 15.78 mg/g, and 48.74 mg/g, respectively. Pumpkin proteins contained 16.24 mg/g and 53.20 mg/g of previously mentioned compounds, respectively. Pea proteins contained only procyanidin B1 at a concentration of 1.83 mg/g, while on the rice protein matrix epicatechin was detected at a concentration of 11.32 mg/g.

Results of polyphenol evaluation and antioxidant activity are presented in Table 3. Almond and pumpkin-seed protein complexes had the highest total polyphenol content, approximately 6.2 mg/g, followed by rice protein complexes with 4.86 mg/g and pea protein complexes with 3.63 mg/g. Almond and pumpkin-seed protein complexes also had the highest proanthocyanidin content, 0.982 and 0.905 mg/g, respectively, while the pea protein complex contained 0.716 mg/g and the rice protein complex 0.412 mg/g. Antioxidant activity values showed a different trend. Various methods were used to evaluate antioxidant activity, resulting in different trends among the samples. The highest antioxidant activity by the CUPRAC method was observed for the almond protein complex, followed by pea, rice, and finally pumpkin-seed protein complexes, with values of 7.951 mmol TE/100 g, 6.644 mmol TE/100 g, 6.059 mmol TE/100 g, and 5.109 mmol TE/100 g, respectively. Results from the FRAP method showed that antioxidant activity was similar for almond and rice protein complexes (approximately 3.1 mmol TE/100 g), followed by pumpkin-seed and pea protein complexes with 2.791 and 2.326 mmol TE/100 g, respectively. According to the DPPH method, almond protein complexes had the highest antioxidant activity of 1.197 mmol TE/100 g, followed by pumpkin-seed and rice protein complexes (approximately 1.05 mmol TE/100 g), while pea protein complexes had the lowest value (0.924 mmol TE/100 g). The ABTS method also indicated that almond protein complexes had the highest antioxidant activity of 2.777 mmol TE/100 g, followed by pumpkin-seed complex (2.499 mmol TE/100 g), rice protein complex (2.294 mmol TE/100 g), and pea protein complex (1.528 mmol TE/100 g).

Concentrations of individual polyphenols determined in protein-based complexes are presented in Table 4. Both anthocyanins that were determined in CPE were also determined in protein-based complexes. Comparing all complexes, there was no statistically significant difference between concentrations of cyanidin-3-galactoside, which were approximately 40.5 µg/g. Regarding cyanidin-3-arabinoside, the highest concentration was measured in the pumpkin protein complex (10.17 µg/g), while the lowest was measured in the pea protein complex (8.96 µg/g). The trend observed in CPE, namely, that the concentration of cyanidin-3-galactoside was higher than that of cyanidin-3-arabinoside, was retained in the complexes. Considering quercetin derivatives, a different trend was observed in protein complexes than in CPE. In addition to the three glycosides detected in CPE, quercetin aglycone was also quantified, probably due to cleavage of the glycosidic bond, resulting in liberation of quercetin. Moreover, while in CPE the concentrations of quercetin derivatives decreased in the order quercetin-3-glucoside > quercetin-3-rutinoside > quercetin-3-galactoside, this was not the case in protein complexes. The highest concentration of quercetin-3-rutinoside was found in the rice protein complexes (524.58 µg/g), followed by quercetin-3-galactoside (25.82 µg/g) and quercetin-3-glucoside (20.91 µg/g). The almond protein complex also had the highest concentration of quercetin-3-rutinoside (330.29 µg/g), followed by quercetin-3-glucoside (63.64 µg/g) and quercetin-3-galactoside (35.38 µg/g). The same tendency was observed in the pea protein complex, i.e., the highest concentration of quercetin-3-rutinoside (215.56 µg/g), followed by quercetin-3-glucoside (48.96 µg/g) and quercetin-3-galactoside (28.22 µg/g). Only the pumpkin protein complex had the highest concentration of quercetin-3-glucoside (66.51 µg/g), followed by quercetin-3-galactoside (54.16 µg/g) and quercetin-3-rutinoside (29.09 µg/g). As already mentioned, protein complexes contained quercetin at concentrations of 110.92 µg/g, 57.18 µg/g, 48.24 µg/g, and 19.35 µg/g for almond, pea, rice, and pumpkin protein complexes, respectively. Regarding phenolic acids, all three that were determined in CPE were also determined in protein complexes. In CPE, the decreasing order of phenolic acid concentrations was neochlorogenic acid > chlorogenic acid > isochlorogenic acid, with neochlorogenic acid considerably higher than the other two, a trend that was not retained in protein complexes. The highest concentration of neochlorogenic acid was in the rice protein complex (373.91 µg/g), followed by chlorogenic acid (300.30 µg/g) and isochlorogenic acid (102.57 µg/g). The same declining trend was observed for almond and pumpkin protein complexes. For the almond protein complex, concentrations were 209.57 µg/g for neochlorogenic acid, 201.39 µg/g for chlorogenic acid, and 77.46 µg/g for isochlorogenic acid, while for the pumpkin protein complex concentrations were 200.52 µg/g, 192.18 µg/g, and 60.97 µg/g, respectively. The pea protein complex was the only one with similar concentrations of neochlorogenic and chlorogenic acids, approximately 167 µg/g, while the concentration of isochlorogenic acid was 64.34 µg/g.

Catechin, epicatechin, and procyanidin B1 were determined in CPE; however, as previously explained, not all of these compounds were detected in protein matrices; thus, not all of them were detected in protein complexes. The almond protein complex contained the highest concentration of procyanidin B1 (21.69 µg/g), followed by the pumpkin (19.41 µg/g) and pea (1.08 µg/g) protein complexes. The pumpkin and almond protein complexes also contained catechin at concentrations of 1.65 µg/g and 0.43 µg/g, respectively, while additionally the pumpkin protein complex contained a low concentration of epicatechin (0.79 µg/g). The rice protein complex was the only one that did not contain these types of phenolic compounds.

**Figure 1:**
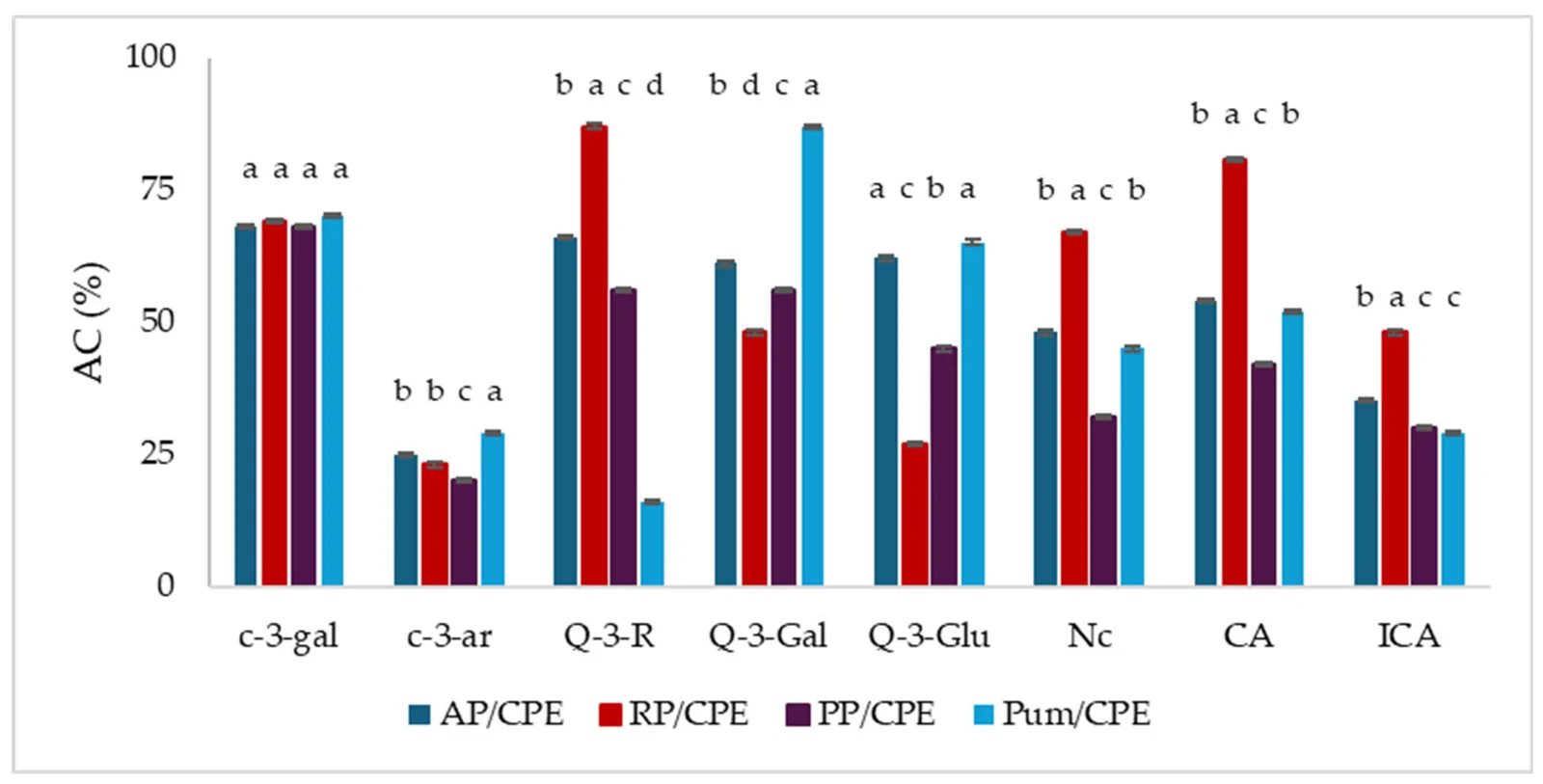
Adsorption capacity (%) of anthocyanins, quercetin derivatives and phenolic acids for protein matrices (CPE—extract of chokeberry pomace, AP—almond proteins, RP—rice proteins, PP—pea proteins, PumP—pumpkin-seed proteins, c-3-gal—cyanidin-3-galactoside, c-3-ar—cyanidin-3-arabinoside, Q-3-R—quercetin-3-rutinoside, Q-3-Gal—quercetin-3-galactoside, Q-3-Glu—quercetin-3-glucoside, Nc—neochlorogenic acid, ICA—isochlorogenic acid, and CA—chlorogenic acid). Data marked for each compound with the different letters (a–d) are significantly different.

Adsorption capacity (Figure 1) was determined for anthocyanins, quercetin derivatives and phenolic acids, as they were not estimated in protein matrices but were determined in CPE and protein-based complexes. The adsorption capacity (AC) for cyanidin-3-galactoside in all protein matrices was around 69%, while considerably lower ACs were found for cyanidin-3-arabinoside. The highest AC for cyanidin-3-arabinoside was observed in pumpkin proteins (29%) and the lowest in rice proteins (23%), while the other two protein matrices had an AC of approximately 25%. Rice proteins had the highest AC for quercetin-3-rutinoside (87%), followed by almond (66%), pea (56%) and pumpkin (16%) protein matrices. For quercetin-3-galactoside, the highest AC was achieved by the pumpkin protein matrix (87%), followed by almond (61%), pea (56%) and rice (48%) protein matrices. For quercetin-3-glucoside, a similar trend was achieved. The highest AC was achieved by the pumpkin protein matrix (65%), followed by almond (62%), pea (45%) and rice (27%) protein matrices. The rice protein matrix also had the highest AC for phenolic acids: 67% for neochlorogenic acid, 81% for chlorogenic acid and 48% for isochlorogenic acid. The almond protein matrix had the highest AC for chlorogenic acid (54%), followed by 48% for neochlorogenic acid and 35% for isochlorogenic acid. The same trend was observed for the pumpkin protein matrix, i.e., AC for chlorogenic acid was 52%, for neochlorogenic acid 45% and for isochlorogenic acid 29%. Chlorogenic acid also exhibited the highest AC on the pea protein matrix, where AC was 42%, with 31% for the other two phenolic acids.

### 2.2. Color Parameters and pH-Dependent Stability

As evident from Table 5, the created complexes vary in their color; thus, color parameters were determined and compared. L* values after adsorption of polyphenols from CPE in protein matrices significantly decreased, ranging from 49.31 to 66.74, with RP/CPE having the highest lightness and PP/CPE the lowest. The other two complexes had the same L* value (52.6). a* values also changed after adsorption of CPE polyphenols, increasing for almond, rice, and pumpkin-seed proteins, while for pea proteins it slightly decreased. The AP/CPE complex had the highest redness (13.08), followed by RP/CPE (7.01). The other two complexes had lower redness: 3.28 and 1.34 for PumP/CPE and PP/CPE, respectively. b* values were significantly higher for protein matrices and decreased after adsorption of polyphenols. The b* parameter was highest for PumP/CPE (6.02), followed by RP/CPE (4.15), PP/CPE (0.87), and AP/CPE (−1.55). The color difference in protein matrices due to adsorption of polyphenols was also calculated; values decreased in the following order: AP/CPE > PP/CPE > PumP/CPE > RP/CPE (37.40 > 35.10 > 26.02 > 20.02).

**Figure 2:**
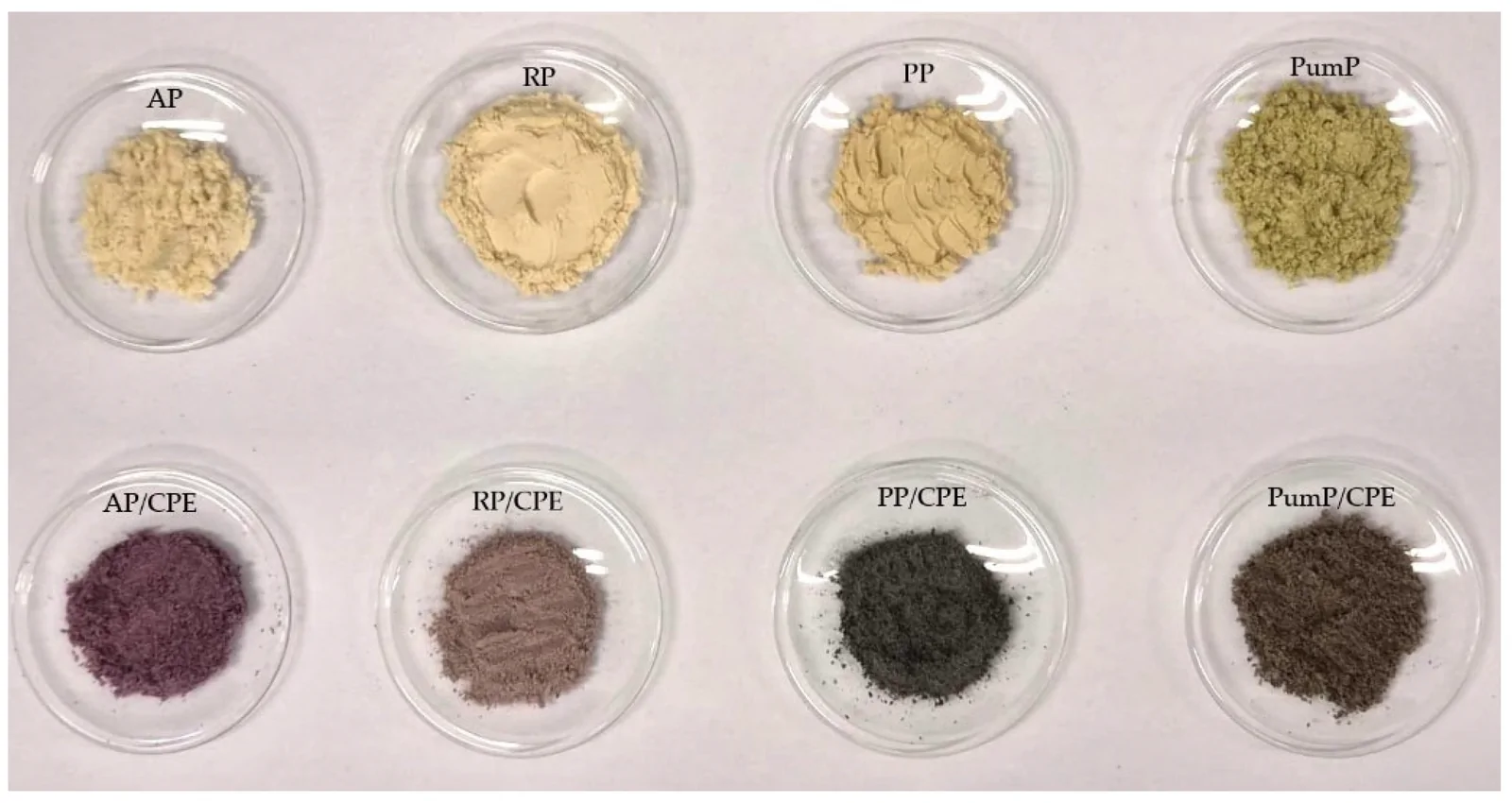
Presentation of selected protein matrices and created complexes (CPE—extract of chokeberry pomace, AP—almond proteins, RP—rice proteins, PP—pea proteins, and PumP—pumpkin-seed proteins).

**Figure 3:**
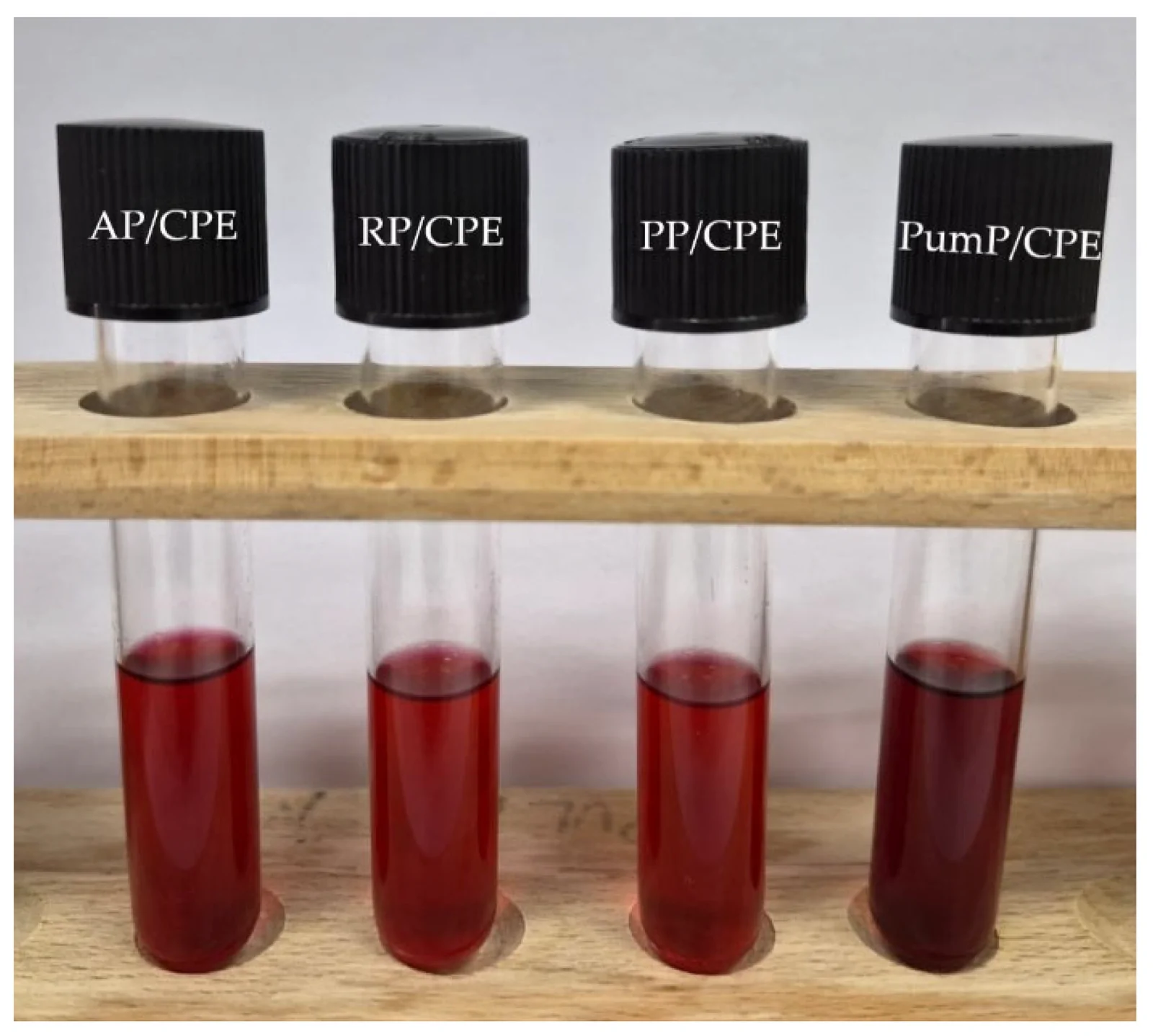
Color of extracts of protein-based complexes (CPE—extract of chokeberry pomace, AP—almond proteins, RP—rice proteins, PP—pea proteins, and PumP—pumpkin-seed proteins).

Protein matrices (first row) and corresponding complexes (second row) are shown in Figure 2. It is evident that the color of the protein matrices changed; however, PP/CPE and PumP/CPE were not violet (AP/CPE) or light violet-gray (RP/CPE) like the other two complexes. This is due to the pH value of the protein matrices. The results of pH measurements are presented in Table 6. pH values were recorded for CPE and for the mixture of CPE with each protein matrix after 15 min of complexation in order to show how the protein matrices affected the pH value of CPE. The pH value of CPE was 3.6. The highest pH value of the protein and CPE mixture was determined for pea protein at 6.24, followed by almond proteins at 5.63, pumpkin at 5.57, and rice proteins at 4.87. It is well known that anthocyanins are highly susceptible to changes in pH and that they are structurally and color stable at lower pH values. The measured pH values indicate changes in the anthocyanin structures; thus, the color of the protein complexes was not red as it was when polyphenols were extracted in acidified methanol (Figure 3).

### 2.3. FTIR-ATR Spectral Analysis

**Figure 4:**
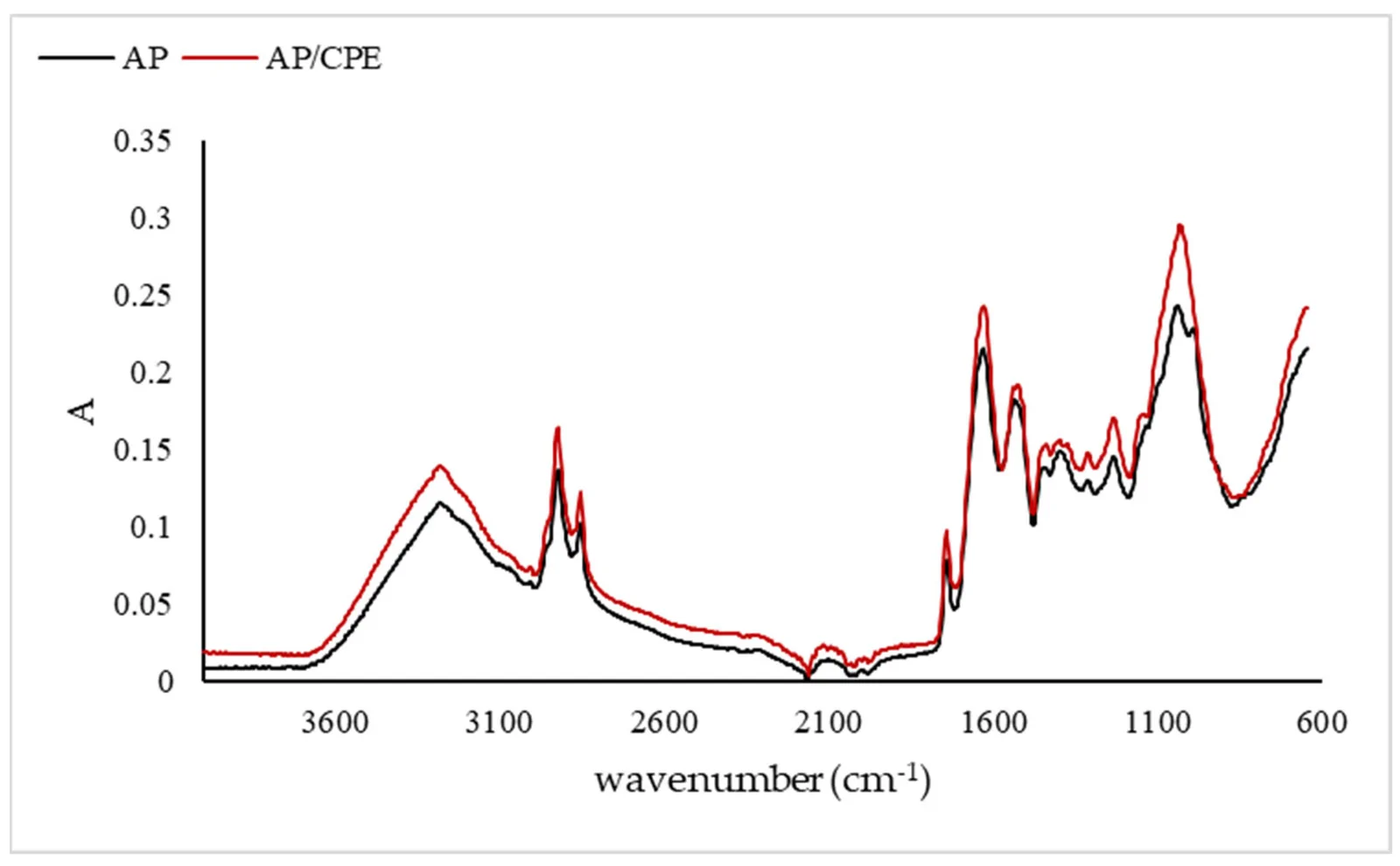
IR spectra of almond protein matrix and created complex (CPE—extract of chokeberry pomace, AP—almond proteins).

**Figure 5:**
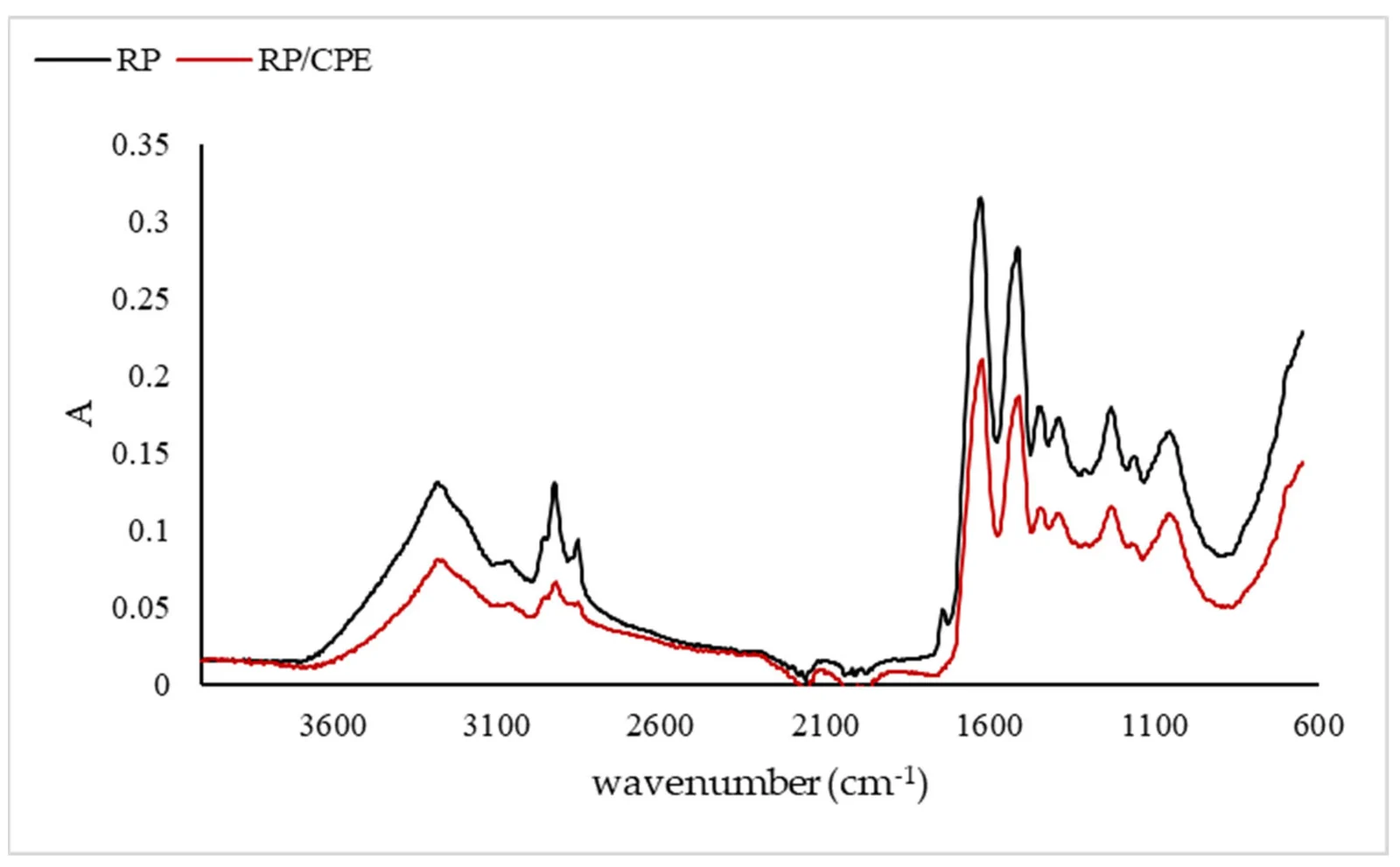
IR spectra of rice protein matrix and created complex (CPE—extract of chokeberry pomace, RP—rice proteins).

**Figure 6:**
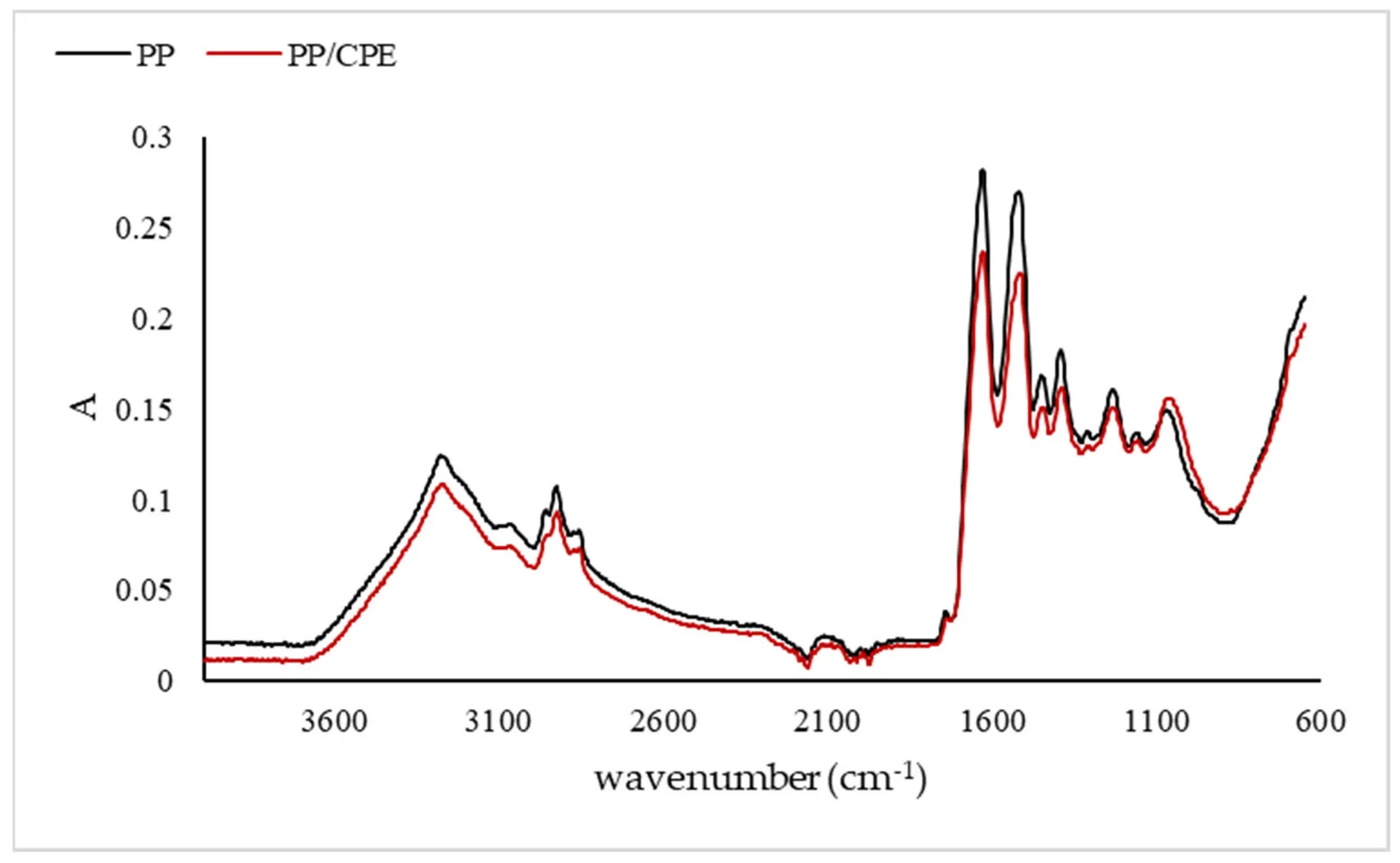
IR spectra of pea protein matrix and created complexes (CPE—extract of chokeberry pomace, PP—pea proteins).

**Figure 7:**
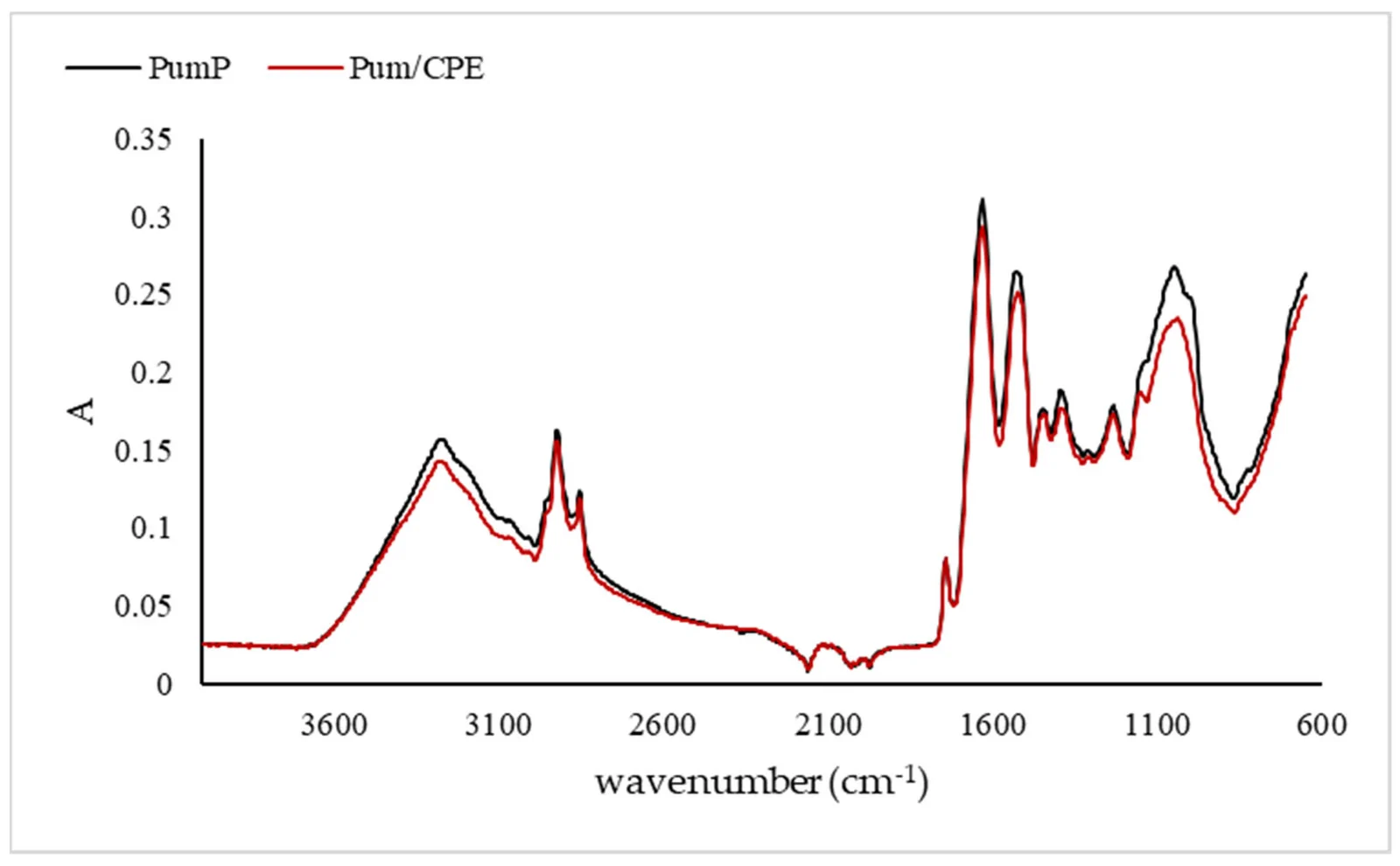
IR spectra of pumpkin-seed protein matrix and created complexes (CPE—extract of chokeberry pomace, PumP—pumpkin-seed proteins).

Results of screening IR spectra of protein matrices and corresponding complexes are presented in Figure 4, Figure 5, Figure 6 and Figure 7. Complexation of polyphenols from CPE on all protein matrices caused changes in protein structures. Protein structures are specific by amide regions, specifically: the region 3300–3500 cm^−1^ which is assigned to amide A (associated with N–H stretching), the region 3100–2980 cm^−1^ assigned to amide B (associated with N–H and C–H stretching), the region 1700–1600 cm^−1^ assigned to amide I (linked to C–O stretching), region 1600–1500 cm^−1^ assigned to amide II (associated with N–H bending and C–H stretching), and the region 1380–1200 cm^−1^ assigned to amide III (associated with N–H in-plane bending coupled with C–N stretching) [13,14,15,16,17].

The most significant change in the almond protein structure (Figure 4) after the adsorption of CPE was observed in the band at 1397 cm^−1^ and in the fingerprint region of polysaccharides. The band at 1397 cm^−1^, associated with symmetric CH_3_ bending of the methyl groups in proteins, became broader. Since almond proteins contained fibers, the fingerprint region of polysaccharides, located in the 1200–900 cm^−1^ range, is highly important. Almond proteins had a band at 1043 cm^−1^ with a slight shoulder at 1144 cm^−1^. Adsorption of CPE polyphenols caused the band to shift to 1032 cm^−1^, with a more pronounced shoulder. The band at 1043 cm^−1^ is associated with C–O stretching and C–O bending of the C–OH and –CH_2_OH groups in polysaccharides. The shoulder of the band is associated with the C–O bond in the hydroxyl group of polysaccharides. Additionally, the band at 995 cm^−1^ in almond proteins, which is associated with C–O and C–C bonds, disappeared after adsorption of polyphenols.

Adsorption of polyphenols from CPE onto rice proteins (Figure 5) caused a significant decrease in IR spectral intensity. There was also a change in the ratio of bands intensities at 2925 cm^−1^ and 2855 cm^−1^, indicating that the interactions between polyphenols and rice proteins were of hydrophobic nature. Additionally, the band at 1740 cm^−1^ disappeared due to the adsorption of CPE polyphenols.

Regarding changes in pea protein structure (Figure 6), in addition to the decrease in spectral intensity, the most significant change was observed at the band at 1069 cm^−1^, which shifted to 1062 cm^−1^ after adsorption of polyphenols from CPE, indicating changes in C–O stretching of the phosphodiester. Additional confirmation of this chemical change was the increased intensity of this shifted band and the loss of the shoulder at 965 cm^−1^ that accompanied the main band.

Pumpkin-seed proteins (Figure 7), like almond proteins, also exhibited change in the fingerprint region of polysaccharides, located in the 1200–900 cm^−1^ range, where the most significant changes occurred. In this region, the protein matrix showed one main band at 1051 cm^−1^ with two shoulders at 1233 cm^−1^ and 998 cm^−1^. After adsorption of polyphenols from CPE, the main band shifted to 1043 cm^−1^, while the shoulder at 998 cm^−1^ disappeared and the other shoulder transformed into a band. Changes in the main band indicated alterations in C–O stretching coupled with C–O bending of the C–OH groups of polysaccharides.

## 3. Discussion

All four protein matrices differ in their structure, which may result in varying affinities towards CPE polyphenols. The major protein fractions in almond proteins are globulin and albumin, with globulin being predominant; together, they account for 88–95% of total almond proteins. Amandin is the main globulin fraction, belonging to the same protein family as soybean glycinin and pea legumin [18]. Similarly, in pea proteins, globulin is the major fraction (65–85%), followed by albumin and small amounts of prolamin and glutelin. Legumin and vicilin that are present in a 2:1 ratio are the main fractions of globulin [19]. The prevailing fraction in rice proteins is glutelins, which also include small amounts of prolamin, albumin, and globulin [20]. Pumpkin-seed protein contains albumin, globulin, glutelin, and prolamin fractions, with globulin (cucurbitin) as the primary fraction, which can be comparable to legumin in peas, glycinin in soybeans, and cruciferin in oilseed rape. In total, 59% of total crude proteins in pumpkin seeds are globulin and albumin fractions [21,22]. Generally, for all these protein matrices, the secondary structure is predominantly composed of β-sheets, followed by α-helices, β-turns, and random coils. Additionally, they are globular proteins whose folded conformation is stabilized by hydrophobic interactions, hydrogen bonding, electrostatic interactions, and disulphide bridges [19,20,21,22,23]. Their structure allows for the formation of hydrogen bonds, hydrophobic, electrostatic, and π-π interactions between polyphenols and free amino and carboxyl functional groups on side chains [24,25,26,27]. Furthermore, π-π interactions can also be a result of the accessibility of aromatic amino acids [27,28].

Except for structural features and properties of proteins, structural features and properties of polyphenols are also important factors that may cause varying affinities of proteins for polyphenols [29]. Consequently, the results of this study indicated that structurally different polyphenols vary in their affinity towards proteins. The most important structural features of polyphenols related to this reactivity are the number and position of hydroxyl groups [30], and many studies have been conducted with the purpose to explore the importance of structural features of proteins and polyphenols on their interactions. Rawel et al. [30] investigated the reactivity and adsorption of polyphenols on soy proteins and found that these proteins had the highest affinity for gallic acid, followed by quercetin and chlorogenic acid, while lower adsorption was observed for caffeic acid, myricetin, and kaempferol. Substantially lower affinity for soy proteins was found for apigenin and flavone. Gallic and chlorogenic acids showed the highest affinity for adsorption onto albumin, a fraction of white bean proteins, while lower affinity was observed for catechin and quercetin, and substantially lower for apigenin and ferulic acid [29]. They also suggested that these polyphenols had different tendencies towards another fraction of white bean proteins, namely globulin. It was determined that affinity decreased in the following order: chlorogenic acid, catechin, gallic acid and quercetin, apigenin, and ferulic acid [29]. In addition to investigating the affinity of individual polyphenols for protein fractions, the same group of authors highlighted the significance of polyphenol composition in complex matrices such as green coffee and green tea extracts. They found that chlorogenic acid and catechin from extracts had higher affinity for the mentioned protein fractions than the pure compounds, indicating that other polyphenols present in the extracts affected their adsorption onto the carrier [29]. A comparison of the adsorption of pomegranate polyphenols on pea and rice proteins, which were also used in this study, revealed that pea proteins had a higher affinity for proanthocyanidins, cyanidin-3-glucoside, delphinidin-3-glucoside, cyanidin-3,5-diglucoside, delphinidin-3,5-diglucoside, ellagic acid, and punicalagin. Gallic acid was the only compound from pomegranate juice that had a higher affinity for rice proteins [31]. In addition, it was found that rice proteins had a higher affinity than pea proteins for total polyphenols and proanthocyanidins when polyphenols from chokeberry juice were adsorbed on them, while the affinity for monomeric anthocyanins was similar for both proteins [32].

The protein matrices used in this study, as defined in Section 4, contain other compounds such as carbohydrates and lipids, which also affect the adsorption of polyphenols. This trend has already been indicated in other studies [33,34,35,36,37]. Investigation of the affinity of cranberry polyphenols towards proteins revealed that their affinity decreased in the following order: defatted soy flour (50% of protein), medium roasted peanut flour (50% of protein), and hemp protein isolate (>70% of protein) [34]. The affinity of blueberry juice anthocyanins decreased in the following order: defatted soy flour (47% of protein), white whole-wheat flour (13% of protein), brown rice flour (8.6% of protein), and corn flour (5.3% of protein) [33]. Comparing the affinity of quercetin towards proteins, it was determined that the almond protein matrix (50% of protein) had a lower affinity for this phenolic compound than brown rice proteins (85% of protein) [35]. The affinity of glucosyl hesperidin for proteins decreased in the following order: pea proteins (85% of protein), almond proteins (50% of protein), brown rice proteins (85% of protein), and pumpkin proteins (50% of protein) [36]. Investigation of the affinity of cinnamic acid towards proteins suggested that not only the type of protein matrix but also its amount affected adsorption. Consequently, when 1% of protein matrix was used for encapsulation of cinnamic acid, the following decrease in affinity was observed: pumpkin proteins (50% of protein), pea proteins (85% of protein), and almond proteins (50% of protein). With an increased amount of protein matrix, a different order was observed: pea proteins (85% of protein), pumpkin proteins (50% of protein), and almond proteins (50% of protein) [37]. All these investigations demonstrated that the type of proteins, their content, and additional organic compounds affected the encapsulation of different types of polyphenols.

The different adsorption of polyphenols on protein matrices also manifested on antioxidant activity of complexes. However, regarding antioxidant activity, the selection of methods is very important. Antioxidant activity is one of the most important properties of polyphenols, and to estimate this property we selected the CUPRAC, FRAP, DPPH and ABTS methods. The antioxidant reactivity of polyphenols strongly depends on their chemical structure; however, additional parameters that can affect antioxidant expression are also important. The concentration of individual polyphenols, the ratio between them, their mutual interactions, and their interactions with other compounds are all highly important. The selected methods are the most frequently used and differ in their mechanisms of action. Whereas the CUPRAC and FRAP methods are based on metal reduction, DPPH and ABTS are based on scavenging free radicals by the antioxidants present. The mechanism of action of CUPRAC is based on the reduction in cupric (Cu^2+^) to cuprous (Cu^+^) ions [38], while the mechanism of action of the FRAP method involves the reduction in the ferric ion (Fe^3+^)–ligand complex to the ferrous (Fe^2+^) complex [39]. In addition, they are performed at different pH values, pH 7 or pH 3.6, respectively. From the results of this study, it is evident that the samples had higher cupric reducing power; however, the FRAP reaction with antioxidants is slower than CUPRAC. Moreover, whereas CUPRAC reacts with both hydrophilic and lipophilic antioxidants, FRAP reacts more with hydrophilic ones [38,39,40]. The DPPH and ABTS methods involve the reaction of hydrogen-atom donors, i.e., antioxidants, with the corresponding free radicals, DPPH˙ and ABTS˙^+^, which are formed during the initial reaction. Despite the similar mechanism, as with the previous two methods, different results were obtained; specifically, the ABTS antioxidant values were higher [41]. Since the radicals are structurally different, their structures govern their reactivity with antioxidants. ABTS˙^+^ free radicals are less selective and can react with any hydroxylated aromatic compounds, regardless of their actual antioxidant capacity, which can lead to reactions with OH groups that do not contribute to antioxidation [42]. In contrast, DPPH˙ free radicals are more selective than ABTS˙^+^. The DPPH˙ radical is relatively large, which hinders its reaction with larger antioxidants. It also reacts with flavonoids that lack OH groups in the structure of their B-ring and does not react with aromatic acids that have only one OH group [42].

One need to have in mind is that different sources of polyphenols can be used for complexation or encapsulation, such as various types of extracts from fruits or fruit pomace obtained by extraction by different type of solvents, or juice which may result in the difference in polyphenol composition and ratio; all the factors that, at the end, affect their encapsulation on carriers and, consequently, antioxidant activity. The presence of different phenolic compounds in the system can cause additive, antagonistic or synergistic effects between them due to the diversity of their structures. As already mentioned, the structure of polyphenols, specifically the number and position of OH groups and OCH_3_ groups on the phenolic rings, is accountable for their antioxidant activity. However, for a total comprehension of the antioxidant potential of multicomponent systems, such as formulated complexes, additional parameters need to be included. In addition to the structure of polyphenols, their concentrations and relative ratio between them, their dissociation and ionization, intramolecular and/or intermolecular interactions, and matrix interference are very important [38,43,44].

As can be seen in Figure 2, not all complexes had red/violet color, which was probably due to changes in anthocyanin structure caused by pH. When in equilibrium, anthocyanins exist in four chemical forms. The flavylium cation is red and dominant at pH 1–2; the carbinol pseudobase is colorless, dominant at pH 3–5, and forms after the flavylium cation is hydrated; the quinoidal anion is blue-purple and forms in the pH range 6–9; and chalcone is pale yellow and forms above pH 9 [45]. Although the pH of CPE was 3.6, after the addition of protein matrices, the pH increased and altered the pH of the complexation mixtures, which consequently changed the structure of the anthocyanins and their color, thus affecting the final color of the complexes. Even though anthocyanin structure changed due to pH, the carbinol pseudobase still can interact with proteins and bind on the surface; thus, when pigments are extracted from complexes, they change their structure back to the flavylium cation, as was evident from Figure 3. The complexation mixture consists mainly of water that combined with pH cause changes on anthocyanins. Water causes a nucleophilic attack on the C-2 position of anthocyanins, which may result in the formation of the carbinol pseudobase. Subsequently, the C-ring of anthocyanins opens to form chalcone, which further deteriorates to a brown product [46,47,48].

During the complexation of proteins and polyphenols, in addition to the interactions between these compounds and the influence of water and pH, an additional mechanism may be involved in the adsorption of CPE polyphenols on proteins. Most likely, a stacking effect—interactions between anthocyanins from CPE and anthocyanins already bonded to protein—can occur [32,49,50,51,52]. Anthocyanins are susceptible to π-stacking interactions with themselves (self-association) and with other polyphenols (especially flavonoids and hydroxycinnamic acids) that can be found together with anthocyanins [51]. This effect may be more pronounced on almond proteins, which remained red despite the high pH. Thus, adsorption is the consequence of several different factors.

The presence of water has to be considered as a factor which has influence on the encapsulation of polyphenols onto proteins. A large amount of water, as it was in CPE, creates conditions that initiate diffusion-dependent reactions. The presence of water increases molecular mobility, which consequently enhances hydrolysis and oxidation reactions. In addition to the previously described structural changes due to pH shifts, hydrolysis of the glycosidic bond of anthocyanins to anthocyanidins, which are more unstable, also may occur, leading to further degradation [53]. Furthermore, the availability of oxygen accelerates the oxidation rate of anthocyanins. The oxidation of anthocyanins also may result in the formation of colorless and brown compounds such as phenolic acids (syringic, vanillic, and protocatechuic acids; in our case protocatechuic acid was the dominant one since samples contained cyanidin derivatives) [54]. In addition, oxidized intermediates can polymerize or condense with other phenolic compounds (such as tannins), changing the color from vibrant hues to dull ones [55].

At the end, instead of freeze-drying to obtain a dry powder of protein-based complexes, drying at 50 °C was used. It is well known that freeze-drying requires expensive equipment and results in substantial costs; therefore, we selected a less costly drying method. Drying temperature strongly affects polyphenol stability, especially anthocyanins. Temperatures above 75 °C cause their thermal degradation; however, moderate temperatures between 50 °C and 65 °C can be a good alternative when high-cost drying methods such as freeze-drying are to be avoided [56,57,58].

## 4. Materials and Methods

### 4.1. Materials

Chokeberry pomace and plant-based proteins were used for the preparation of protein complexes. Preparation of chokeberry extract is described in the following section. Regarding plant-based proteins, four of them were selected. The almond proteins matrix (approximately 53% of proteins; 12% of fats; and 7% of carbohydrates) and rice protein matrix (approximately 85% of proteins; 5% of fats; and 7.7% of carbohydrates (from which 2% were fibers)) were obtained from Biovega (Zagreb, Croatia); the pea protein powder (approximately 85% of proteins; 8.7% of fats; 2.5% of carbohydrates (from which 1.9% of fibers)) was obtained from Biesterfeld Spezialchemie d.o.o. (Zagreb, Croatia), and pumpkin-seed meal protein powder (approximately 55% of proteins; 12% of fats; 17% of carbohydrates (from which 11% of fibers)) was obtained from Nutrigold (Zagreb, Croatia).

For spectrophotometric and HPLC evaluation of protein complexes, the following chemicals and standards were used. From Carlo Erba Reagents (Sabadell, Spain) we obtained methanol and hydrochloric acid (37%), while acetic acid (>99.5%) was obtained from Alkaloid (Skopje, North Macedonia). Gram-mol (Zagreb, Croatia) was the supplier for ethanol, ammonium acetate, sodium acetate, calcium chloride and potassium chloride while T.T.T. (Sveta Nedelja, Croatia) was the supplier for sodium carbonate. From Kemika (Zagreb, Croatia) we bought Folin–Ciocalteu reagent and potassium persulfate, while neocuproine (>99%), 2,4,6-tri(2-pyridyl)-s-triazine (99%), and cupric chloride were products of Acros Organic (Geel, Belgium). Sigma-Aldrich (St. Louis, MO, USA) was the supplier for 2,2′-azino-bis(3-ethylbenzothiazoline-6-sulphonic acid) diammonium salt (>98%), 2,2-diphenyl-1-picrylhydrazyl, 4-dimethylaminocinnamaldehyde, Trolox (97%), procyanidn B1, procyanidin B2 (>90%), chlorogenic acid, catechin, (-)-epicatechin, quercetin-3-rutinoside, quercetin-3-rutinoside, quercetin-3-galactoside, quercetin-3-glucoside and quercetin from, while cyanidin-3-galactoside, neochlorogenic acid, and gallic acid (>97%) were obtained from Extrasynthese (Genay, France). Orthophosphoric acid (HPLC grade) we obtained from Fisher Scientific (Loughborough, UK) and methanol (HPLC grade) from J.T. Baker (Deventer, The Netherlands).

### 4.2. Ultrasound-Assisted Extraction of Chokeberry Pomace

Pomace remaining after chokeberry juice production was dried in an oven at 55 °C until a constant mass was achieved. The dried chokeberry pomace was grinded and then extracted with acidified water (distilled water adjusted to pH 4.5 with acetic acid) in an ultrasonic bath (Bandelin, Berlin, Germany) for 30 min at room temperature, with a nominal ultrasonic power of 80 W and ultrasonic frequency of 35 kHz. The ratio between chokeberry pomace and acidified water was 1:20. After ultrasonication, the mixture was centrifuged at 8000 rpm for 15 min at room temperature, and the supernatant was collected; thus, chokeberry pomace extract (CPE) was obtained and used for encapsulation with plant-based proteins.

### 4.3. Preparation of Protein Complexes

Each protein matrix, 2 g, was complexed with 20 mL of CPE, and each combination was prepared twice. Complexation was performed by agitation in a glass on a magnetic stirrer at room temperature for 15 min. After complexation, the mixture was transferred to a plastic tube and centrifuged for 15 min at 8000 rpm at room temperature. The supernatant was then separated from the precipitate. The precipitate was dried in an oven at 50 °C for 5 h until a constant mass was achieved, while the supernatant was used to estimate adsorption capacity as described in the following section.

### 4.4. Ultrasound-Assisted Extraction of Protein Complexes

Ultrasound-assisted extraction was conducted to extract polyphenols from protein complexes. In a plastic tube, 0.6 g of protein complex was weighed and mixed with 7 mL of acidified methanol (methanol: concentrated HCl 99:1), which was used as the extraction solvent. The tubes were placed in an ultrasonic bath (Bandelin, Berlin, Germany) for 30 min at room temperature, with a nominal ultrasonic power of 80 W and ultrasonic frequency of 35 kHz. After ultrasonication, the mixture was centrifuged at 8000 rpm for 15 min at room temperature, and the supernatant was collected and used as the extract for spectrophotometric determinations and HPLC analysis.

### 4.5. Assessment of Total Polyphenols and Procyanidins

The contents of total polyphenols and procyanidins were determined spectrophotometrically using a UV-Vis spectrophotometer (Cary 60, Agilent Technologies, Santa Clara, CA, USA). Total polyphenols were assessed using the Folin–Ciocalteu method as specified by Singleton and Rossi [59], while procyanidins were assessed using the DMAC method specified by Prior et al. [60]. Each experiment was performed in triplicate, with results expressed as mg of gallic acid equivalents per g of complex (mg GAE/g) and mg of procyanidin B2 equivalents per g of complex (mg PB2E/g), respectively.

### 4.6. Qualitative and Quantitative Assessment of Individual Polyphenols

Individual polyphenols were qualitatively and quantitatively assessed by HPLC analysis. For this purpose, a 1260 Infinity II HPLC system (Agilent Technology, Santa Clara, CA, USA) equipped with a quaternary pump, vial sampler, and two detectors, a diode array detector (DAD) and a fluorescence detector (FLD), was used. For compoun separation, a Poroshell 120 EC C-18 column (4.6 × 100 mm, 2.7 µm) was employed. Before injection onto the column, 2 mL of extract was filtered through PTFE filters with a 0.45 µm pore size and then injected at a volume of 10 µL, with a flow rate of 1.0 mL/min. Two mobile phases were used for elution of polyphenols through the column: mobile phase A, orthophosphoric acid (0.1% aqueous solution), and mobile phase B, methanol (100%). The elution gradient used for compound separation was as follows: 0 min, 5% B; 3 min, 30% B; 15 min, 35% B; 22 min, 37% B; 30 min, 41% B; 32 min, 45% B; 40 min, 49% B; 45 min, 80% B; 48 min, 80% B; 50 min, 5% B; and 53 min, 5% B.

For anthocyanins, phenolic acids, and flavonoids, the DAD detector was used, and UV-Vis spectra were scanned from 190 nm to 600 nm. The FLD detector was used for procyanidin detection, with fluorescence measured at an excitation wavelength of 278 nm and an emission wavelength of 360 nm.

Standards were used for identifying polyphenols through comparison with sample chromatograms for retention times, while for anthocyanins, phenolic acids and flavonoids, and, additionally, UV-Vis spectra, were also compared. Anthocyanins were measured at 520 nm, phenolic acids at 280 nm, and flavonoids at 360 nm. Calibration curves were used for compound quantification, and results were expressed as µg of compound per g of sample (µg/g). A calibration curve for cyanidin-3-galactoside was prepared in the range from 1 to 500 mg/L (r^2^ = 0.9998; LOD = 0.0006 mg/L; LOQ = 0.0019 mg/L), for cyanidin-3-arabinoside from 1 to 150 mg/L (r^2^ = 0.9996; LOD = 0.29 mg/L; LOQ = 0.89 mg/L), for quercetin-3-rutinoside from 5 to 550 mg/L (r^2^ = 0.998; LOD = 2.12 mg/L; LOQ = 6.44 mg/L), for quercetin-3-galactoside from 2.5 to 125 mg/L (r^2^ = 1; LOD = 0.18 mg/L; LOQ = 0.55 mg/L), for quercetin-3-glucoside from 2.5 to 200 mg/L (r^2^ = 0.9997; LOD = 0.20 mg/L; LOQ = 0.60 mg/L), for quercetin from 1 to 150 mg/L (r^2^ = 0.9998; LOD = 0.605 mg/L; LOQ = 1.83 mg/L), for neochlorogenic acid from 5 to 1500 mg/L (r^2^ = 0.9992; LOD = 0.71 mg/L; LOQ = 2.15 mg/L), and for chlorogenic acid from 5 to 1000 mg/L (r^2^ = 0.9997; LOD = 0.0002 mg/L; LOQ = 0.0006 mg/L), for catechin from 0.5 to 20 mg/L (r^2^ = 0.9995; LOD = 0.79 mg/L; LOQ = 1.05 mg/L), for (-)-epicatechin from 0.5 to 20 mg/L mg/L (r^2^ = 0.9992; LOD = 0.15 mg/L; LOQ = 0.3 mg/L), for procyanidin B1 from 1 to 100 mg/L (r^2^ = 0.9998; LOD = 0.18 mg/L; LOQ = 2.76 mg/L).

### 4.7. Estimation of Adsorption Capacity

Adsorption capacity was calculated for anthocyanins, phenolic acids and flavonoids according to the following equation:

AC (%) = ((C_EM_ − C_S_)/C_EM_) × 100

where AC represents the adsorption capacity, and C_EM_ and C_S_ represent the concentrations of each compound assessed by HPLC in the complexation mixture and supernatant, respectively.

### 4.8. Assessment of Antioxidant Activity

The four spectrophotometric methods were selected to assess antioxidant activity, namely CUPRAC, FRAP, DPPH, and ABTS. A UV-Vis spectrophotometer (Cary 60, Agilent Technologies, Santa Clara, CA, USA) was used for measurement of absorbance. Each experiment was conducted in triplicate, with results expressed as mmol of Trolox equivalents per 100 g of complex (mmol TE/100 g). All selected methods were previously described: the CUPRAC method by Apak et al. [61], the FRAP by Benzie and Strain [62], the DPPH method by Brand-Williams et al. [63], and the ABTS method by Arnao et al. [64].

### 4.9. Assessment of pH Values

pH values of CPE and mixtures of CPE with each protein matrix after complexation at room temperature for 15 min were recorded using a pH meter (Orion, Thermo Fisher Scientific, Waltham, MA, USA).

### 4.10. Assessment of IR Spectral Changes

IR spectral changes in protein matrices upon adsorption of CPE were assessed using the FTIR-ATR instrument (Cary 630, Agilent, Santa Clara, CA, USA), equipped with MicroLab Expert software (Agilent, Santa Clara, CA, USA). Samples (protein matrices and corresponding complexes) were placed on the ATR and pressed. IR spectra were recorded at room temperature in the range 4000–600 cm^−1^, with a resolution of 4 cm^−1^ and collection of 32 scans.

### 4.11. Assessment of Color Parameters

Color parameters were assessed using a Minolta CR-400 chromameter (Konica Minolta, Inc., Osaka, Japan). The CIELAB color space was used for expression of color parameters by recording L* (lightness), a* (redness (+) and greenness (−)), and b* (yellowness (+) and blueness (−)). Based on the recorded L*, a*, and b* values, the total color difference (∆E) between complexes and the corresponding protein matrix was calculated. All measurements were performed in triplicate.

### 4.12. Statistical Assessment of Results

Results were presented as mean values ± standard deviation Statistical assessment of the experimental results was done by STATISTICA 13.1 (StatSoft Inc., Tulsa, OK, USA). The results were evaluated by analysis of variance (ANOVA), followed by Fisher’s least significant difference (LSD) test to determine significant differences at *p* < 0.05.

## 5. Conclusions and Future Perspective

Chokeberry pomace is a rich but underutilized source of polyphenols. Plant-based proteins and chokeberry pomace extract on their own exhibited antioxidant activity due to the present compounds; thus, the final antioxidant potential of the protein complexes was the result of compounds in the pure protein matrix and polyphenols adsorbed from chokeberry juice in the protein matrices. Overall, almond and pumpkin protein complexes exhibited the highest antioxidant potential, but it should be kept in mind that antioxidant potential is the result of all present compounds and their concentrations and ratios, as well as their interactions. Considering individual compounds, all protein complexes had the same concentration of cyanidin-3-galactoside, while the pumpkin protein complex had a slightly higher concentration of cyanidin-3-arabinoside. The rice protein complex contained a considerably higher concentration of quercetin-3-rutinoside and all phenolic acids. Pumpkin protein complexes had the highest concentrations of quercetin-3-galactoside and quercetin-3-glucoside, while almond had the highest concentration of quercetin. Pumpkin and almond protein complexes had the highest concentrations of procyanidin B1. The results of this study contribute to the field of polyphenol–protein interactions, highlighting the importance of the structure and properties of both polyphenols and proteins. Also, when using plant-based protein matrices, the presence of other compounds has to be taken into account, as can be seen from this study: they behave differently and contain different protein contents.

For future investigations, these results provide a good baseline for the formulation of protein-based complexes using chokeberry pomace rich in polyphenols. However, this research can be expanded to different fruit pomace extracts, as well as other plant-based proteins or fiber sources. These complexes can be used in food products to enhance their antioxidant potential or to enrich those products with both types of compounds. However, additional studies should be conducted, including evaluations of their storage stability, incorporation into food products, controlled release, and the behavior of these compounds in the human body through investigations of in vitro digestion and bioaccessibility.

## Figures and Tables

**Table 1:** Total polyphenol (Tpc) and procyanidins (Pc) contents (mg/g) and antioxidant activities (mmol TE/100 g) of chokeberry pomace extract (CPE) and protein matrices.

	Tpc	Pc	CUPRAC	FRAP	DPPH	ABTS
CPE *	0.769 ± 0.047	5.910 ± 0.099 ^a^	10.987 ± 0.075 ^a^	5.897 ± 0.074 ^a^	2.413 ± 0.031 ^a^	3.891 ± 0.458 ^a^
AP	-	0.318 ± 0.013 ^c^	3.417 ± 0.528 ^b^	0.740 ± 0.039 ^b^	0.424 ± 0.026 ^b^	0.264 ± 0.001 ^c^
RP	-	0.078 ± 0.001 ^d^	1.742 ± 0.035 ^c^	0.187 ± 0.007 ^d^	0.468 ± 0.011 ^b^	0.028 ± 0.005 ^d^
PP	-	0.480 ± 0.017 ^b^	4.436 ± 0.628 ^b^	0.652 ± 0.221 ^c^	0.291 ± 0.015 ^c^	0.038 ± 0.006 ^d^
PumP	-	0.272 ± 0.046 ^c^	1.152 ± 0.084 ^d^	0.719 ± 0.019 ^b^	0.447 ± 0.022 ^b^	0.338 ± 0.046 ^b^

AP—almond proteins, RP—rice proteins, PP—pea proteins, and PumP—pumpkin-seed proteins. *—results for CPE, for Tpc presented as g/L, for Pc as mg/L and for antioxidant activity as mmol TE/100 mL. Data marked in the column with the different letters (a–d) are significantly different.

**Table 2:** Individual polyphenol concentrations of chokeberry pomace extract (CPE) and protein matrices.

	CPE (mg/L)	AP (mg/g)	RP (mg/g)	PP (mg/g)	PumP (mg/g)
c-3-gal	92.08 ± 0.80	-	-	-	-
c-3-ar	31.09 ± 0.37	-	-	-	-
Q-3-R	124.72 ± 0.34	-	-	-	-
Q-3-Gal	109.94 ± 0.21	-	-	-	-
Q-3-Glu	155.29 ± 0.61	-	-	-	-
Nc	1181.28 ± 1.93	-	-	-	-
ICA	129.07 ± 0.31	-	-	-	-
CA	731.05 ± 1.14	-	-	-	-
Cat	4.71 ± 0.09	15.78 ± 0.09	-	-	16.24 ± 0.16
EpiC	8.12 ± 0.13	-	11.32 ± 0.19	-	-
Pr B1	5.59 ± 0.83	48.74 ± 0.18	-	1.83 ± 0.11	53.20 ± 0.21

AP—almond proteins, RP—rice proteins, PP—pea proteins, PumP—pumpkin-seed proteins, c-3-gal—cyanidin-3-galactoside, c-3-ar—cyanidin-3-arabinoside, Q-3-R—quercetin-3-rutinoside, Q-3-Gal—quercetin-3-galactoside, Q-3-Glu—quercetin-3-glucoside, Nc—neochlorogenic acid, ICA—isochlorogenic acid, CA—chlorogenic acid, Cat—catechin, EpiC—(-)-epicatechin, and Pr B1—procyanidin B1.

**Table 3:** Total polyphenol (Tpc) and proanthocyanidins (Pac) contents (mg/g) and antioxidant activities (mmol TE/100 g) of protein-based complexes.

	Tpc	Pac	CUPRAC	FRAP	DPPH	ABTS
AP/CPE	6.339 ± 0.191 ^a^	0.982 ± 0.021 ^a^	7.951 ± 0.275 ^a^	3.095 ± 0.285 ^a^	1.197 ± 0.032 ^a^	2.777 ± 0.040 ^a^
RP/CPE	4.856 ± 0.058 ^b^	0.412 ± 0.008 ^d^	6.059 ± 0.163 ^c^	3.247 ± 0.261 ^a^	1.064 ± 0.018 ^b^	2.294 ± 0.023 ^c^
PP/CPE	3.634 ± 0.022 ^b^	0.716 ± 0.023 ^c^	6.644 ± 0.344 ^b^	2.326 ± 0.035 ^c^	0.924 ± 0.015 ^c^	1.528 ± 0.099 ^d^
PumP/CPE	6.058 ± 0.191 ^a^	0.905 ± 0.022 ^b^	5.109 ± 0.190 ^d^	2.791 ± 0.019 ^b^	1.033 ± 0.015 ^b^	2.499 ± 0.060 ^b^

CPE—extract of chokeberry pomace, AP—almond proteins, RP—rice proteins, PP—pea proteins, and PumP—pumpkin-seed proteins. Data marked in the column with the different letters (a–d) are significantly different.

**Table 4:** Individual polyphenol concentrations (µg/g) in protein-based complexes.

	AP/CPE	RP/CPE	PP/CPE	PumP/CPE
c-3-gal	40.73 ± 0.80 ^a^	40.68 ± 0.78 ^a^	39.52 ± 0.81 ^a^	40.99 ± 0.79 ^a^
c-3-ar	9.48 ± 0.11 ^b^	9.09 ± 0.09 ^bc^	8.96 ± 0.08 ^c^	10.17 ± 0.09 ^a^
Q-3-R	330.29 ± 0.89 ^b^	524.58 ± 2.14 ^a^	215.56 ± 1.08 ^c^	29.09 ± 0.75 ^d^
Q-3-Gal	35.38 ± 0.29 ^b^	25.82 ± 0.87 ^d^	28.22 ± 0.59 ^c^	54.16 ± 0.99 ^a^
Q-3-Glu	63.64 ± 0.56 ^b^	20.91 ± 0.49 ^d^	48.96 ± 0.52 ^c^	66.51 ± 0.58 ^a^
Q	110.92 ± 0.78 ^a^	48.24 ± 0.75 ^c^	57.18 ± 0.69 ^b^	19.35 ± 0.43 ^d^
Nc	209.57 ± 0.92 ^b^	373.91 ± 1.18 ^a^	166.05 ± 1.29 ^d^	200.52 ± 1.28 ^c^
ICA	77.46 ± 0.74 ^b^	102.57 ± 0.93 ^a^	64.34 ± 0.91 ^c^	60.97 ± 0.87 ^d^
CA	201.39 ± 0.83 ^b^	300.30 ± 1.15 ^a^	167.39 ± 1.71 ^d^	192.18 ± 1.09 ^c^
Cat	0.43 ± 0.09 ^b^	-	-	1.65 ± 0.13 ^a^
EpiC	-	-	-	0.79 ± 0.03 ^a^
Pr B1	21.69 ± 0.83 ^a^	-	1.08 ± 0.09 ^c^	19.41 ± 0.43 ^b^

CPE—extract of chokeberry pomace, AP—almond proteins, RP—rice proteins, PP—pea proteins, PumP—pumpkin-seed proteins, c-3-gal—cyanidin-3-galactoside, c-3-ar—cyanidin-3-arabinoside, Q-3-R—quercetin-3-rutinoside, Q-3-Gal—quercetin-3-galactoside, Q-3-Glu—quercetin-3-glucoside, Q—quercetin, Nc—neochlorogenic acid, ICA—isochlorogenic acid, CA—chlorogenic acid, Cat—catechin, EpiC—(-)-epicatechin, Pr B1—procyanidin B1. Data marked in the row with the different letters (a–d) are significantly different.

**Table 5:** Color parameters of protein matrices and protein-based complexes.

	L*	a*	b*	ΔE
AP	83.53 ± 0.01 ^a^	0.96 ± 0.01 ^g^	15.83 ± 0.01 ^d^	
AP/CPE	52.71 ± 0.17 ^f^	13.08 ± 0.06 ^a^	−1.44 ± 0.01 ^h^	37.4
RP	75.41 ± 0.02 ^c^	4.57 ± 0.01 ^c^	22.03 ± 0.03 ^a^	
RP/CPE	66.74 ± 0.02 ^e^	7.01 ± 0.01 ^b^	4.15 ± 0.00 ^f^	20.02
PP	78.82 ± 0.06 ^b^	2.92 ± 0.01 ^e^	19.89 ± 0.05 ^c^	
PP/CPE	49.31 ± 0.16 ^g^	1.34 ± 0.02 ^f^	0.87 ± 0.01 ^g^	35.10
PumP	71.85 ± 0.16 ^d^	−2.11 ± 0.04 ^h^	22.65 ± 0.10 ^b^	
PumP/CPE	52.58 ± 0.21 ^f^	3.28 ± 0.01 ^d^	6.02 ± 0.04 ^e^	26.02

CPE—extract of chokeberry pomace, AP—almond proteins, RP—rice proteins, PP—pea proteins, PumP—pumpkin-seed proteins. ΔE—color change in protein complex in comparison with corresponding protein matrix. Data marked in the column with the different letters (a–h) are significantly different.

**Table 6:** pH values of CPE and mixture of CPE and each protein matrix.

Samples	pH
CPE	3.60 ± 0.01 ^e^
Mixture of AP and CPE	5.63 ± 0.01 ^b^
Mixture of RP and CPE	4.87 ± 0.01 ^d^
Mixture of PP and CPE	6.24 ± 0.01 ^a^
Mixture of PumP and CPE	5.57 ± 0.01 ^c^

CPE—extract of chokeberry pomace, AP—almond proteins, RP—rice proteins, PP—pea proteins, PumP—pumpkin-seed proteins. Data marked in the column with the different letters (a–e) are significantly different.

## Data Availability

The original contributions presented in this study are included in the article. Further inquiries can be directed to the corresponding author.
